# Long-term major events after hospital discharge for out-of-hospital cardiac arrest

**DOI:** 10.1186/s13613-024-01371-6

**Published:** 2024-09-12

**Authors:** Sofia Ortuno, Wulfran Bougouin, Sebastian Voicu, Marine Paul, Jean-Baptiste Lascarrou, Sarah Benghanem, Florence Dumas, Frankie Beganton, Nicole Karam, Eloi Marijon, Xavier Jouven, Alain Cariou, Nadia Aissaoui

**Affiliations:** 1 Service de Médecine Intensive Réanimation, Université de ParisHôpital Européen Georges Pompidou, AP-HP, Paris, France; 2 Service de Médecine Intensive Réanimation, Hôpital Privé Jacques Cartier, Ramsay Générale de Santé After-ROSC Network, INSERM U970, Paris Sudden-Death- Expertise-Center, Massy, France; 3 Service de Réanimation Médicale et Toxicologique, Hôpital Lariboisière, AP-HP INSERM UMRS-1144 Paris Université de Paris, Paris, France; 4 Service de Médecine Intensive Réanimation, After-ROSC Network Hôpital André Mignot Université de Paris, Versailles, France; 5 Service de Médecine Intensive Réanimation, CHU Nantes After-ROSC Network INSERM U970, Sudden Death Expertise Center, Paris, France; 6 Service de Médecine Intensive Réanimation, Hôpitaux Universitaires Paris, Hôpital Cochin, AP- HP Paris, Université de Paris, 27 Rue du Faubourg Saint-Jacques, Paris, 75014 France; 7 Service d’urgences, Hôpitaux Universitaires Paris, Hôpital Cochin, AP-HP Paris Sudden Death Expertise Center Université de Paris, Paris, France; 8 Département de Cardiologie, Hôpital Européen Georges Pompidou, AP-HP INSERM U970 Sudden Death Expertise Center, Paris, France; 9 After-ROSC Network, INSERM U970 INSERM UMRS - 1144 Paris Sudden-Death- Expertise-Center, Paris, France

**Keywords:** Cardiac arrest, Long-term outcomes, Cardiovascular outcome, Out-of-hospital cardiac arrest, Death

## Abstract

**Background:**

Cardiac arrest remains a global health issue with limited data on long-term outcomes, particularly regarding recurrent cardiovascular events in patients surviving out-of-hospital cardiac arrest. (OHCA). We aimed to describe the long-term occurrence of major cardiac event defined by hospital admission for cardiovascular events or death in OHCA hospital survivors, whichever came first. Our secondary objective were to assess separately occurrence of hospital admission and death, and to identify the factors associated with major event occurrence. We hypothesized that patients surviving an OHCA has a protracted increased risk of cardiovascular events, due to both presence of the baseline conditions that lead to OHCA, and to the cardiovascular consequences of OHCA induced acute ischemia-reperfusion.

**Methods:**

Consecutive OHCA patients from three hospitals of Sudden Death Expertise Center (SDEC) Registry, discharged alive from 2011 to 2015 were included. Long-term follow-up data were obtained using national inter-regime health insurance information system (SNIIRAM) database and the national French death registry. The primary endpoint was occurrence of a major event defined by hospital admission for cardiovascular events and death, whichever came first during the follow-up. The starting point of the time-to-event analysis was the date of hospital discharge. The follow-up was censored on the date of the first event. For patients without event, follow-up was censored on the date of December, 29th, 2016.

**Results:**

A total of 306 patients (mean age 57; 77% male) were analyzed and followed over a median follow-up of 3 years for hospital admission for cardiovascular event and 6 years for survival. During this period, 38% patients presented a major event. Hospital admission for cardiovascular events mostly occurred during the first year after the OHCA whereas death occurred more linearly during the all period. A previous history of chronic heart failure and coronary artery disease were independently associated with the occurrence of major event (HR 1.75, 95%CI[1.06-2.88] and HR 1.70, 95%CI[1.11-2.61], respectively), whereas post-resuscitation myocardial dysfunction, cardiogenic shock and cardiologic cause of cardiac arrest did not.

**Conclusion:**

Survivors from OHCA must to be considered at high risk of cardiovascular event occurrence whatever the etiology, mainly during the first year following the cardiac arrest and should require closed monitoring.

**Supplementary Information:**

The online version contains supplementary material available at 10.1186/s13613-024-01371-6.

## Background

Out-of-hospital cardiac arrest (OHCA) is a major public health problem occurring in approximately 41 people per 100,000 persons-year in Europe and 47 people per 100,000 persons-year in North America [1]. Short-term outcome is very poor, less than 10% of patients being alive at hospital discharge [2–4]. Among the patients discharged alive from hospital, long term outcome is also a concern. A recent meta-analysis assessing data of 11,800 individual survivors at hospital discharge from of 21 studies reported a 16.8% mortality rate on a median of follow-up of 5 years [5]. This previous metanalysis focused on long-term survival rate of patients who survived the initial hospital stay for an out-of-hospital cardiac arrest without analysing occurrence of cardiovascular events after hospital discharge. Most studies assessing long-term morbidities of these patients focused on neurologic disabilities and recurrence of rhythmic events [6].

Considering that main causes of cardiac arrest are of cardiac origin, particularly acute coronary artery disease (60–80%) [7], and that up to 50% of OHCA patients suffer of post resuscitation shock with myocardial dysfunction at the early phase of ICU care, it is surprising that only a few studies assessed the long-term occurrence of cardio-vascular events in OHCA survivors [5]. The few assessing predictors of such cardiovascular events reported high mortality rate mainly related to cardiac disease [8, 9].

In this study, we aimed to describe the long-term occurrence of major cardiac event defined by hospital admission for cardiovascular events or death in OHCA hospital survivors, whichever came first. Our secondary objective was to assess separately occurrence of hospital admission and death, and last to identify the factors associated with major event occurrence.

We hypothesized that patients surviving an OHCA has a protracted increased risk of cardiovascular events, due to both presence of the baseline conditions that lead to OHCA, and to the cardiovascular consequences of OHCA induced acute ischemia-reperfusion.

## Patients and methods

### Study design and data source

We conducted a retrospective multicenter study including patients from the Sudden Death Expertise Center (SDEC) Registry. The SDEC Registry consists of a population-based registry, covering the area of Paris (France) and its suburbs, representing a residential population of approximately 6.6 million, described in detail previously [10]. French appropriate review boards approved the registry: Commission Nationale de l’Informatique et des Libertés (CNIL) approval #912,309, and Comité Consultatif sur le Traitement de l’Information en matière de Recherche dans le domaine de la Santé (CCTIRS) approval #12,336. The study was performed according to the 2013 Declaration of Helsinki of the World Medical Association regarding medical investigations.

### Study participants

Among the SDEC registry population, patients admitted for OHCA in one of the three university hospitals (Lariboisière, European Georges Pompidou and Cochin hospitals) were screened for inclusion. All patients discharged alive from these three hospitals after OHCA between May 2011 to November 2015 were included. Patients were excluded only if no long-term follow-up data were available. Of note, the 3 university hospitals meet the criteria regarding expert centers in the management of cardiac arrest as defined by international guidelines [11].

### Endpoints

The primary endpoint was the occurrence a major event, defined as a composite criterion combining hospital admission for cardiovascular event and death from any cause occurring during the period of follow-up.

Hospital admission for cardiovascular event included all acute coronary, cerebrovascular or other atheroma related vascular events and heart failure requiring hospital admission. For the primary endpoint, we considered only the first event (hospital admission for cardiovascular event or death) that occurred during the follow-up whichever comes first.

Secondary endpoints were the occurrence of hospital admission for cardiovascular event, the occurrence of death from any cause and prescription of drugs recommended in patients with heart failure recorded at hospital discharge and within the first year [12].

Then, we try to identify factors associated with the occurrence of major event.

### Data collection

Data included baseline characteristics, intensive care unit (ICU) management and long-term follow-up.

Baseline characteristics included demographic data (age, sex, comorbidities, and cardiovascular risk factors) and pre-hospital management including location of arrest, presence of witnessed arrest, bystander cardiopulmonary resuscitation (CPR), initial shockable cardiac arrest rhythm, defibrillation attempts, use of epinephrine. Regarding the ICU period, data regarding vasopressors and inotropes, target temperature management (defined by therapeutic hypothermia at 33 °C, or normothermia at 36 °C if not tolerated), coronary angiography, identification of a cardiac cause of arrest, initial left ventricle ejection fraction (LVEF at ICU admission). Cardiac cause of cardiac arrest included complicated coronary occlusion, primary or secondary arrythmia, complications of dilated, hypertrophic or valvular cardiomyopathy or all other congenital heart disease and tamponade. We also recorded the occurrence of post-resuscitation shock, defined by either systolic blood pressure < 90mmHg or mean arterial pressure < 65mmHg, or requiring vasopressors to maintain MAP > 65mmHg due to vasoplegic shock, occurrence of cardiogenic shock defined by an alteration of LVEF at admission requiring inotropic treatment, occurrence of post-resuscitation myocardial dysfunction (LVEF at ICU admission lower then 40%), hospital length of stay, neurological outcome at hospital discharge, LVEF at hospital discharge, and the guidelines-directed medical therapy in heart failure (including angiotensin converting enzyme inhibitor or mineralocorticoid receptor antagonist, diuretic and betablocker) at hospital discharge and within the first year. Good neurological outcome was defined as a Cerebral Performance Category Scale (CPC) score 1 and 2, i.e., patients independent for activities of daily living, evaluated at hospital discharge. Most data were collected from the SDEC registry where data regarding ICU evolution and hospital discharge were retrospectively collected.

Long-term follow-up data regarding cardiovascular events were obtained using national inter-regime health insurance information system (SNIIRAM) database. SNIIRAM is a national large encoded database including all information related to health insurance reimbursement linked to the national hospital-discharge summaries database system and the national death registry [10, 13, 14]. It is a part of the national system of health information, created in 1998 to evaluate expenditure of all health insurance, assess health policy and improve quality of care, covers 98.8% of the French population [13, 14]. Information collected concerned the insured and detailed coding (CIM 10) about treatment delivered, medical acts, medical devices and biological samples. Thus, it allows to identify all care resources used including medications, procedures, and hospitalizations and collect long-term data regarding hospital admission for cardiovascular event [13]. Of note, the use of SNIIRAM data required authorizations from the Consultative Committee on Data Processing for Health Research (CCTIRS), (reference C12_08), the Institute for Health Data (IDS) and an endorsement by the National Commission for Information Technology and Civil Liberties (CNIL) (reference 912309v2).

Survival was collected from the national French death registry, the Centre for Epidemiology on Medical Causes of Death [CépiDc] registry (cepidc.inserm.fr). The CépiDc registry recorded all death of any person who dies within the geopolitical territory of France. It has been storing French death certificates at the national scale since 1968 and manages a database currently containing nearly 18 million records covering the entire French territory. The collection is exhaustive for the general population but does not include deaths that occurred abroad. Cause of death (cardiovascular or not) was not available for the study period.

### Statistical analysis

Continuous variables were expressed as medians with interquartile range (IQR) or means with standard deviation (SD), depending on distribution, and were compared using Mann-Whitney test or Student’s T-test, as appropriate. We checked the linearity of continuous variables using fractional polynomial regression, and we dichotomized non-linear continuous variables based on the median. Categorical variables were reported as frequencies and percentages, and were compared using the chi-square test.

Occurrence of endpoints were described and expressed in event. The starting point of the time-to-event analysis was the date of hospital discharge. The follow-up was censored on the date of the first event (first hospital admission for cardiovascular event or death). For patients without event, follow-up was censored on the date of December, 29th, 2016. The occurrence of major event was also assessed according to subgroups : patients with or without history of cardiovascular disease, patients with or without cardiac cause of OHCA, patients with CPC 1,2 or CPC 3,4 at hospital discharge.

Survival curves were constructed using the Kaplan-Meier method and compared using the log-rank test. Variables with a P-value less than 0.15 in the univariable analysis were included in a multiple variable model, using a backward selection based on the likelihood-ratio test, to determine the independent effect of patients’ characteristics on the risk of event. The Cox proportional hazards regression analysis was used to identify independent predictors of events. For each variable included, a proportional hazards assumption was tested graphically and verified with Schoenfeld residual (all predictors satisfied the proportional hazard assumption). Analysis was performed with alpha risk of 5%.

We used STATA software, version 15.1 (Lakeway Drive, TX).

## Results

**Fig. 1:**
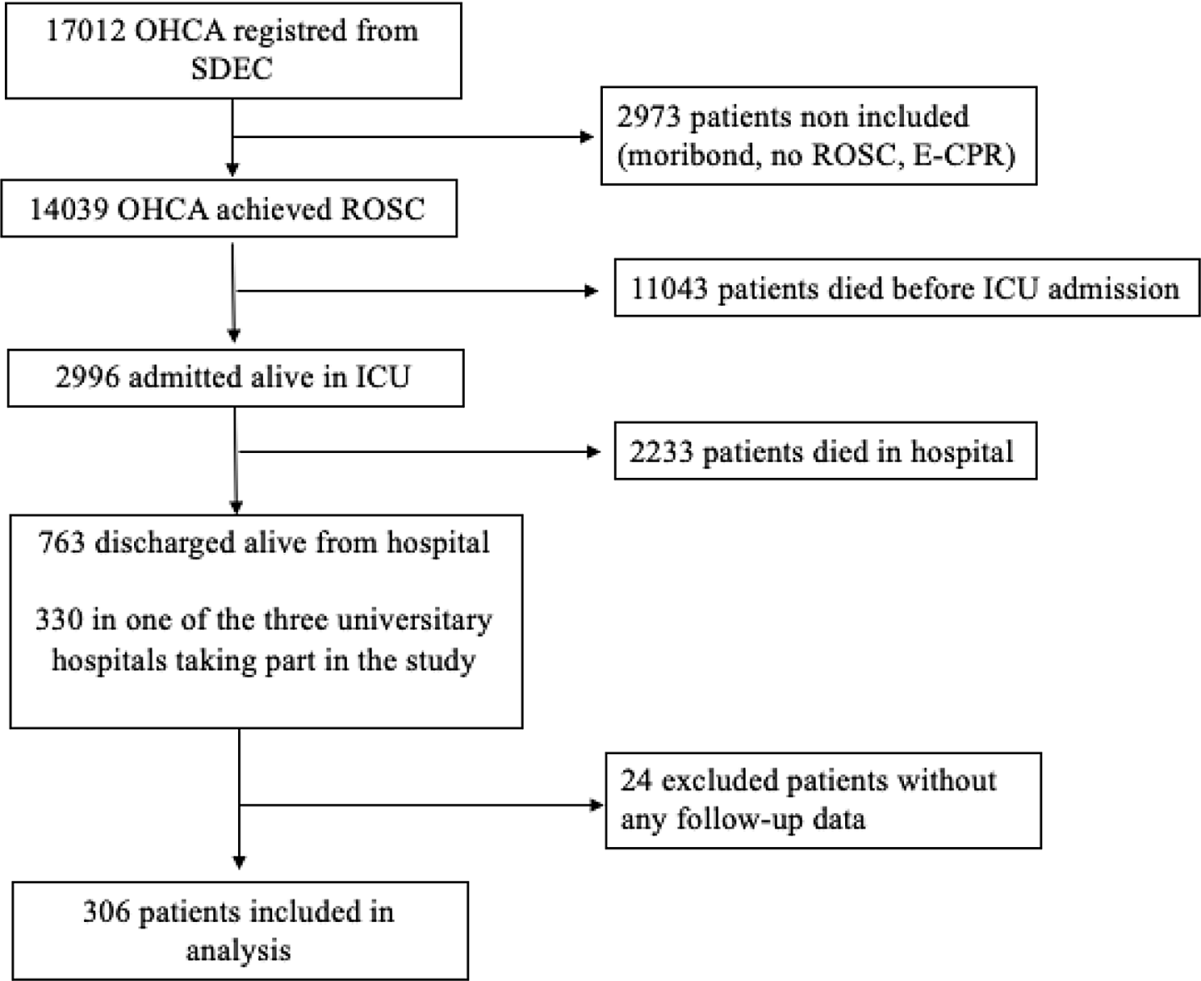
Flow-Chart. Detail patients including in the analysis and reason for exclusion. OHCA : Out-of-Hospital Cardiac Arrest; SDEC: Sudden Death Expertise Center; ROSC: Return Of Spontaneous Circulation; E-CPR : Extra-corporeal-Membrane-Oxygenation associated to Cardio-Pulmonary-Resuscitation; ICU: Intensive Care Unit Of note, among 306 patients included in analysis, CeDC data were not available for 33 patients

Between May 2011 and November 2015, among 17,012 OHCA patients registered in SDEC, 763 OHCA patients were discharged alive from hospital and 330 from one of the three participating hospitals. After excluding patients without any follow-up data (*n* = 24), 306 patients were included in this analysis. Of note, no data regarding CépiDC were available for 33 patients. (Fig. 1).

### Baseline characteristics and ICU management

Baseline patients’ characteristics are presented in Table 1. Among these 306 OHCA hospital survivors, 235 (77%) patients were male, 57 (19%) patients had previous coronary artery disease and 35 (12%) had previous history of chronic heart failure. Two hundred fifty-one of them (83%) presented cardiac arrest with initial shockable rhythm.

**Table 1:** Baseline characteristics and ICU management

Characteristics	Total *N* = 306
**Baseline characteristics**	
Age (years), mean (SD)	57 (16)
Body mass index (kg/m2), mean (SD)	26 (7)
Male, n (%)	235 (77)
History of coronary artery disease, n (%)	57 (19)
History of chronic heart failure, n (%)	35 (12)
Diabetes, n (%)	36 (12)
Dyslipidemia, n (%)	79 (26)
Hypertension, n (%)	100 (33)
Chronic renal failure, n (%)	8 (3)
Chronic Obstructive Pulmonary Disease, n (%)	19 (6)
Smoking, n (%)	174 (58)
Familial history of sudden death, n (%)	30 (11)
**Initial CPR**	
Occurrence at home, n (%)	97 (32)
Witnessed arrest, n (%)	290 (95)
Bystander CPR, n (%)	226 (75)
Initial shockable rythm, n (%)	251 (83)
Collapse to CPR time (minutes), median (IQR)	2 (0–5)
Duration of resuscitation from CPR (minutes), median (IQR)	14 (10–20)
External electric shock count (number), median (IQR)	2 (1–4)
Cumulative dose of epinephrine administrated during CPR (mg), median (IQR)	0 (0–1)
**Initial ICU management**	
Continuous infusion of catecholamine prior to ICU admission, n (%)	139 (45)
Target temperature management, n (%)	244 (80)
Emergency coronary angiography, n (%)	276 (90)
Stenting during primary percutaneous coronary intervention, n (%)	124 (43)
Cardiologic cause of cardiac arrest, n (%)	238 (78)
First LVEF at ICU admission (% LVEF), median (IQR)	40 (30–50)
**ICU evolution**	
Post-resuscitation shock, n (%)	154 (50)
Cardiogenic shock, n (%)	67 (22)
Post-resuscitation myocardial dysfunction, n (%)	153 (50)
Extra corporeal life support for refractory shock, n (%)	2 (1)
Extra-renal replacement therapy, n (%)	44 (14)
Length of mechanical ventilation (days), median (IQR)	4 (2–8)
Length of hospital stay (days), median (IQR)	17 (12–25)
Good neurological outcome at hospital discharge (CPC 1–2), n (%)	260 (85)
**TTE follow-up**	
Last LVEF recorded before hospital discharge (% LVEF), median (IQR)	50 (40–60)
**Drugs recommended in heart failure at hospital discharge**	
Angiotensin-Converting Enzyme inhibitor, n (%)	170 (56)
Beta-blocker, n (%)	174 (57)
Mineralocorticoid receptor antagonist, n (%)	23 (8)
Diuretic, n (%)	79 (26)

CPR: cardiopulmonary resuscitation; CPC: cerebral performance category; ICU: intensive care unit; TTE: transthoracic echocardiography; LVEF: left ventricular ejection fraction; SD: standard deviation; IQR: interquartile range

Regarding initial management, 276 (90%) patients had emergency coronary angiography and 124 patients (43%) benefited from stenting. Cardiac cause of arrest was retained in 238 patients (78%). Post-resuscitation myocardial dysfunction occurred in 153 patients (50%), and cardiogenic shock in 67 patients (22%).

Median length of hospital stay was 17 days (IQR [12–25] days). Two hundred sixty (85%) patients had a good neurological outcome at hospital discharge. Median LVEF at hospital discharge was 50% (IQR [40–60]%). At hospital discharge, regarding guidelines-directed medical therapies in heart failure, 170 patients (56%) had angiotensin-converting enzyme inhibitor, 174 (57%) beta-blocker and 79 (26%) diuretics.

### Primary endpoint

**Fig. 2:**
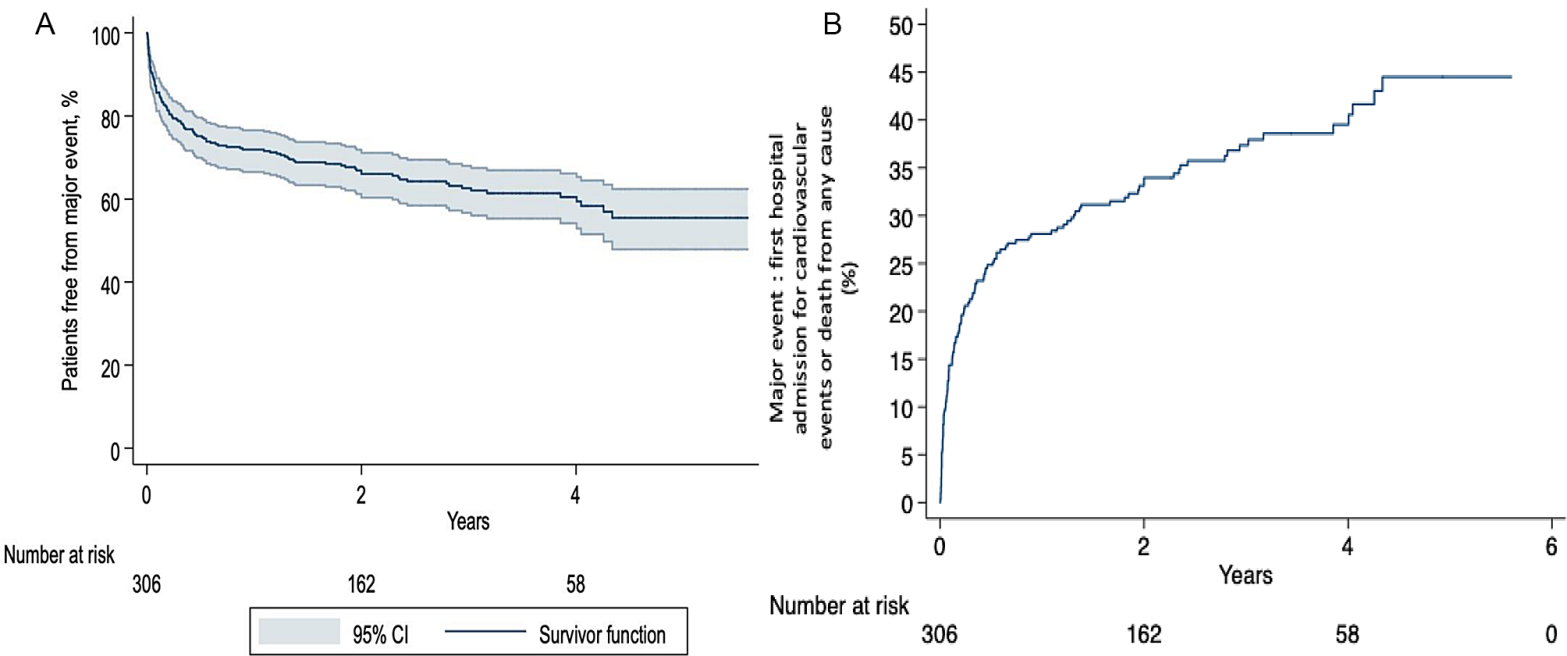
Occurrence of major event, Kaplan-Meier representation. **A**. Shown is Kaplan-Meier estimates of patients free from major events over time. **B**. Shown is Kaplan-Meier estimates of long-term outcome (i.e., composite factor including hospital admission for cardiovascular events or deaths) occurring during the follow-up period, expressed in event per patient-year. The starting point of the time-to-event analysis was the date of hospital discharge of survivors after OHCA

Median length of follow-up was 6 years for survival and 3 years for all other hospital admission for cardiovascular event. Over this follow-up, 116 patients (38%) presented the primary endpoint, either hospital admissions for cardiovascular event or death from any cause, whichever came first: death in 19 patients and hospital admission in 97 patients. Thus, the rate of the primary endpoint (MACE or death) was 16.9% per-patient-year (95% CI: 14.1 − 20.2%). Figure 2A and B depicted patients free from major events over time and occurrence over time of major event.

### Secondary endpoints

**Fig. 3:**
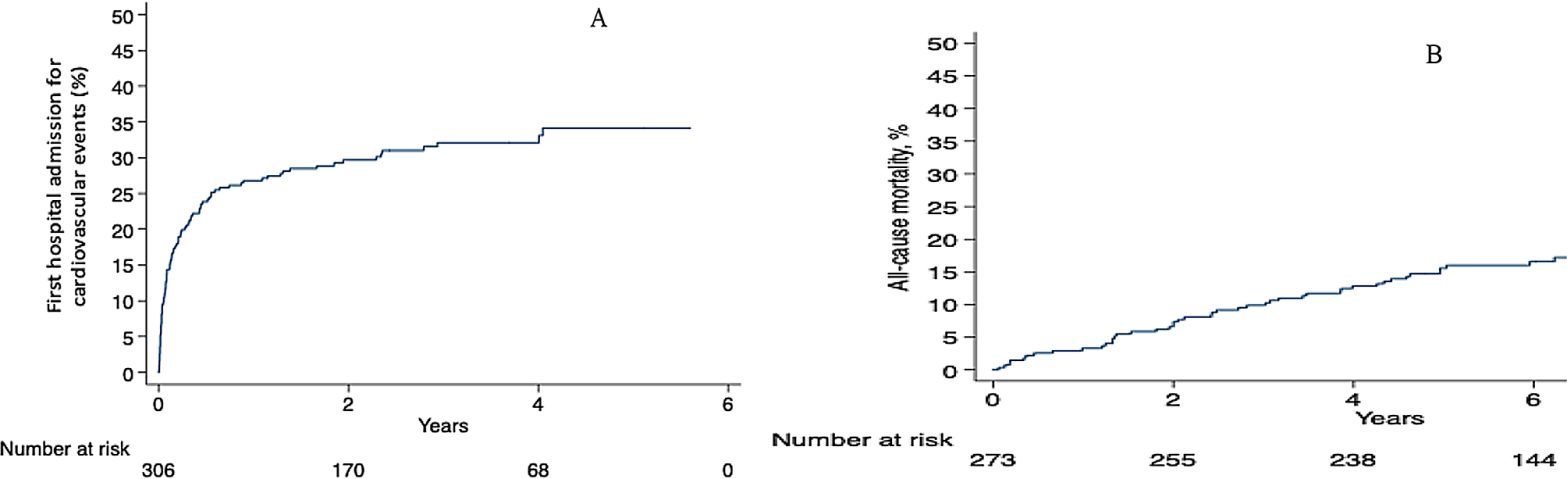
Secondary endpoint, Kaplan-Meier representation. **A**. Shown is Kaplan-Meier estimates of hospital admission for cardiovascular events occurring during the follow-up period, expressed in event per patient-year. **B**. Shown is Kaplan-Meier estimates of deaths from any cause occurring during the follow-up period, expressed in event per patient-year. For both, the starting point of the time-to-event analysis was the date of hospital discharge of survivors after OHCA

Figure 3A shows the occurrence of the first admission for cardiovascular event over the follow-up, and Fig. 3B shows occurrence of death over the follow-up. At 3 years after hospital discharge, 32% of patients followed up had been admitted for a cardiovascular event at least once. At 6 years after hospital discharge, 17% of patients followed up were deceased. Hospital admission for cardiovascular events mostly occurred during the first year after OHCA, unlike deaths which were evenly distributed over the follow-up period. The rate of death from any cause was 3.1% per patient-year (95%CI [2.3–4.1] %) whereas the rate of cardiovascular events excluding death was 13.4% per patient-year (95% CI: 11.0 − 16.4%).

Of note, the 97 patients that were recorded with hospital admission during the follow-up underwent 121 events requiring hospital admission. Main reason was acute heart failure in 52 admissions, acute coronary events in 46, cerebrovascular events in 15, and other cardiovascular events in 8. Among these 97 patients, 23 died during the follow-up period.

One year after hospital discharge, 221 of patients still received drugs recommended for heart failure treatment. Of note, diuretics was prescribed in 108 (35%) patients, 175 (57%) patients received an angiotensin-converting enzyme inhibitor, and 199 (65%) betablocker.

### Factors associated with the occurrence of major event

In univariable analysis, previous history of chronic heart failure and coronary artery disease, initial shockable rhythm and drugs for the treatment of heart failure at one year after cardiac arrest, were associated with occurrence of a major event, see Table 2. The occurrence of post-resuscitation myocardial dysfunction and occurrence of cardiogenic shock were not associated with an increased risk of major event during the follow-up. Neurological status at hospital discharge (evaluated with CPC score) was also not associated with occurrence of major event. Figures depicting KM curves stratified by patient subgroups (e.g. patients with and without chronic heart failure, coronary artery disease, and cardiac cause of OHCA and neurological statuts at hospital discharge) are reported in electronical supplemental material.

**Table 2:** Univariate analysis, factors associated to the occurrence of major event

	Occurrence of major event (hospital admission for cardiovascular events or death) *N* = 116	No major event *N* = 190	*p*
**Baseline characteristics**			
Age > 55, n (%)	66 (57)	89 (47)	0.058
History of coronary artery disease, n (%)	33 (29)	24 (13)	0.001
History of chronic heart failure, n (%)	21 (18)	14 (7)	0.003
**Initial CPR**			
Occurrence at home, n (%)	33 (28)	64 (34)	0.54
Bystander witnesses, n (%)	112 (97)	178 (94)	0.34
Bystander CPR, n (%)	82 (71)	144 (77)	0.29
Collapse to CPR > 2 min, n (%)	49 (44)	78 (44)	0.86
Duration of resuscitation from CPR > 15 min, n (%)	38 (35)	75 (41)	0.37
Cumulative dose of epinephrin administrated during CPR > 2 mg, n (%)	18 (16)	21 (11)	0.28
Cardiologic cause of cardiac arrest, n (%)	95 (82)	143 (75)	0.21
Initial shockable rythm, n (%)	101 (89)	150 (80)	0.047
**Initial and ICU management**			
Primary percutaneous coronary intervention, n (%)	106 (91)	170 (89)	0.54
Stenting during primary percutaneous coronary intervention, n (%)	45 (41)	79 (45)	0.41
Target temperature management, n (%)	91 (79)	153 (81)	0.74
Post-resuscitation myocardial dysfunction (LVEF ≤ 40%), n (%)	60 (61)	93 (57)	0.41
Cardiogenic shock, n (%)	28 (24)	39 (21)	0.36
Shock during ICU stay, n (%)	57 (49)	97 (51)	0.86
Drugs recommended in heart failure, at one year after cardiac arrest . Angiotensin converting enzyme inhibitor or mineralocorticoid receptor antagonist, n (%) . Beta-blocker, n (%) . Diuretic, n (%)	77 (66) 89 (77) 59 (51)	98 (52) 110 (58) 49 (26)	0.008 0.001 < 0.0001
One treatment at least at one year after cardiac arrest, n (%)	89 (77)	132 (69)	0.26
Last LVEF before hospital discharge > 50%, n (%)	22 (39)	46 (49)	0.16
Good neurological outcome, n (%)	100 (38)	160 (84)	0.44

CPR: cardiopulmonary resuscitation; LVEF: left ventricular ejection fraction; ICU: intensive care unit

In multivariable analysis, only previous history of chronic heart failure and coronary artery disease were independently associated with major event occurrence (HR 1.75, 95% CI: [1.06–2.88] ; *p* = 0.027 and HR 1.70, 95% CI: [1.11–2.61] ; *p* = 0.014, respectively).

.

## Discussion

The present work is the largest multicenter longitudinal study assessing long-term cardiovascular outcomes in 306 survivors of out-of-hospital cardiac arrest, with a median follow-up of 6 years for survival and 3 years for all other hospital admission for cardiovascular event. Among these patients, 38% (i.e., 116 patients) were recorded over this follow up with occurrence of a major cardiovascular event i.e., hospital admission for cardiovascular event or death, whichever came first. At 3 years after hospital discharge, 32% of patients followed up had been admitted for a cardiovascular event at least once. At 6 years after hospital discharge, 17% of patients followed up were deceased. Hospital admission for cardiovascular event occurred mostly during the first year after cardiac arrest, unlike deaths which were more evenly distributed over time. Previous history of chronic heart failure and coronary artery disease were both independently associated with occurrence of a major event whereas post-resuscitation myocardial dysfunction, cardiogenic shock and cardiologic cause of arrest did not.

Few recent studies assessed cardiovascular outcome after cardiac arrest and reported similar results [8, 9]. Rey et al. followed a cohort of 201 OHCA patients with a median of 40.3 months and reported that 53 patients (26.4%) presented major adverse cardiovascular events, mainly hospitalization for acute heart failure, stroke and acute myocardial infarction [9]. In a large cohort of 163,071 patients admitted for acute myocardial infarction, Vallabhajosyula et al. followed 12,186 cardiac arrest complicating myocardial infarction patients during 23.5 ± 21.7 months and reported a 31.8% (95%CI [31.5–32.1]) risk of presenting major adverse cardiac and cerebral event [15]. As in our cohort, most of these events occurred during the first year of follow. In our study, the rate of death from any cause was 3% per patient-year (95%CI [2 − 4%]) and 17% were died at 6 years post discharge. This result is consistent with previous reported long-term mortality rates [5, 16, 17]. Indeed, Rey et al. have recently found, in their cohort of 201 OHCA survivors, that 17.9% of patient died during the following period of 40 months [9]. In the same way, a meta-analysis shown that 77.0% patients survived at 5 years after cardiac arrest [5].

Regarding factors associated with long-term major events after OHCA, our study showed that previous cardiac comorbidities were associated with long term cardiovascular and vital events. Our results confirm and extend the results of two recent Canadian and French cohorts that also showed the impact of age and pre-existing comorbidities on outcome [18, 19].

By contrast, it should be noted that most admission for cardiovascular events were in the year following the OHCA, indicating that the factors occurring around the OHCA may be indeed at stake in the development of future cardiovascular events. Of note, when considering patients presenting ACS without cardiac arrest, a recent patient-level meta-analysis of randomized trials assessing 24,325 patients with established CAD, the authors reported a decreased incidence of a primary outcome of cardiovascular death, myocardial infarction, and stroke of 5.9% [20]. All cause death occured in 3.2% of patients. Of note the primary outcome was assessed at a median follow-up time of 493 days [IQR: 369–732 days].

Actually, data we reported on the acute event around the cardiac arrest (i.e., occurrence of post-resuscitation shock, origin the cardiac arrest…) were not associated with an increased risk of major event. Post-resuscitation shock shares some pathophysiologic mechanisms with septic shock [21] and recent data had suggested that septic shock is associated with an increased risk of cardiovascular disease, such as it was expected an association between these events occurring around the resuscitation phase and long term event occurrence. Thus, although we can hypothesize a link between acute phase events and long-term cardiovascular events, the data we registered in this study failed to refine this link. Of note, 221 (85%) patients received drugs recommended in heart failure in our cohort.

Our study presents several limitations. It has a retrospective design, leading to missing data and many patients were lost to follow-up over time. Causes of death were not available in CepiDc registry for the studying period, which could have been particularly interesting to analyze in the 49 patients in which the first event recorded was the decease. Indeed, these deaths might or not be the cause cardiovascular disease, which may change the global picture of the link between OHCA and cardiovascular disease. We were also not able to provide details regarding the history of heart failure, LVEF before admission and during the follow up, to establish a better understanding on the evolution of the cardiac disease from before to after OHCA. Indeed, the SNIIRAM does not allow us to have results from echocardiography. Notably, the median follow-up duration was 6 years for survival outcomes and 3 years for hospital admissions related to cardiovascular events. This discrepancy may have led to a misinterpretation of the results concerning the assessment of hospital admissions for cardiovascular events. Our study could also suffer from the limited number of patients included as only patients from 3 centers where included. However, our study remains one of the largest and the most comprehensive to date.

## Conclusions

Hospital admissions for cardiovascular events and deaths occurred in 38% of patients in the years following resuscitated OHCA. Notably, hospital admissions for cardiovascular events occurred mostly in the first year after cardiac arrest. Our dataset failed to establish a refined link between OHCA and this increased medium-term risk; notably, no link between post-resuscitation shock and major events was observed.

Survivors of cardiac arrest should be considered at high cardiovascular risk regardless of the etiology, especially in the year following the cardiac arrest.

## Electronic supplementary material

Below is the link to the electronic supplementary material.

Supplementary Material 1

## Data Availability

Not applicable.

## Abbreviations

CCTIRS: Comité Consultatif sur le Traitement de l’Information en matière de Recherche dans le domaine de la Santé
CNIL: Commission Nationale de l’Informatique et des Libertés
CPC score: Cerebral Performance Category score
CPR: Cardio-pulmonary Resuscitation
ICU: Intensive Care Unit
LVEF: Left Ventricle Ejection Fraction
OHCA: Out of Hospital Cardiac Arrest
SDEC: Sudden Death Expertise Center
SNIIRAM: National Inter-Regime Health Insurance Information System
