# Supplementary material for: Long-term major events after hospital discharge for out-of-hospital cardiac arrest
Source: Ann Intensive Care. 2024 Sep 12;14:144. doi: 10.1186/s13613-024-01371-6 (PMC11393243; doi:10.1186/s13613-024-01371-6)

**Electronical supplemental material. Occurrence of major event according to supbgroups. Kaplan Meier representation.**

*A. Occurrence of major event according to patients with and without history of coronary artery disease*

**
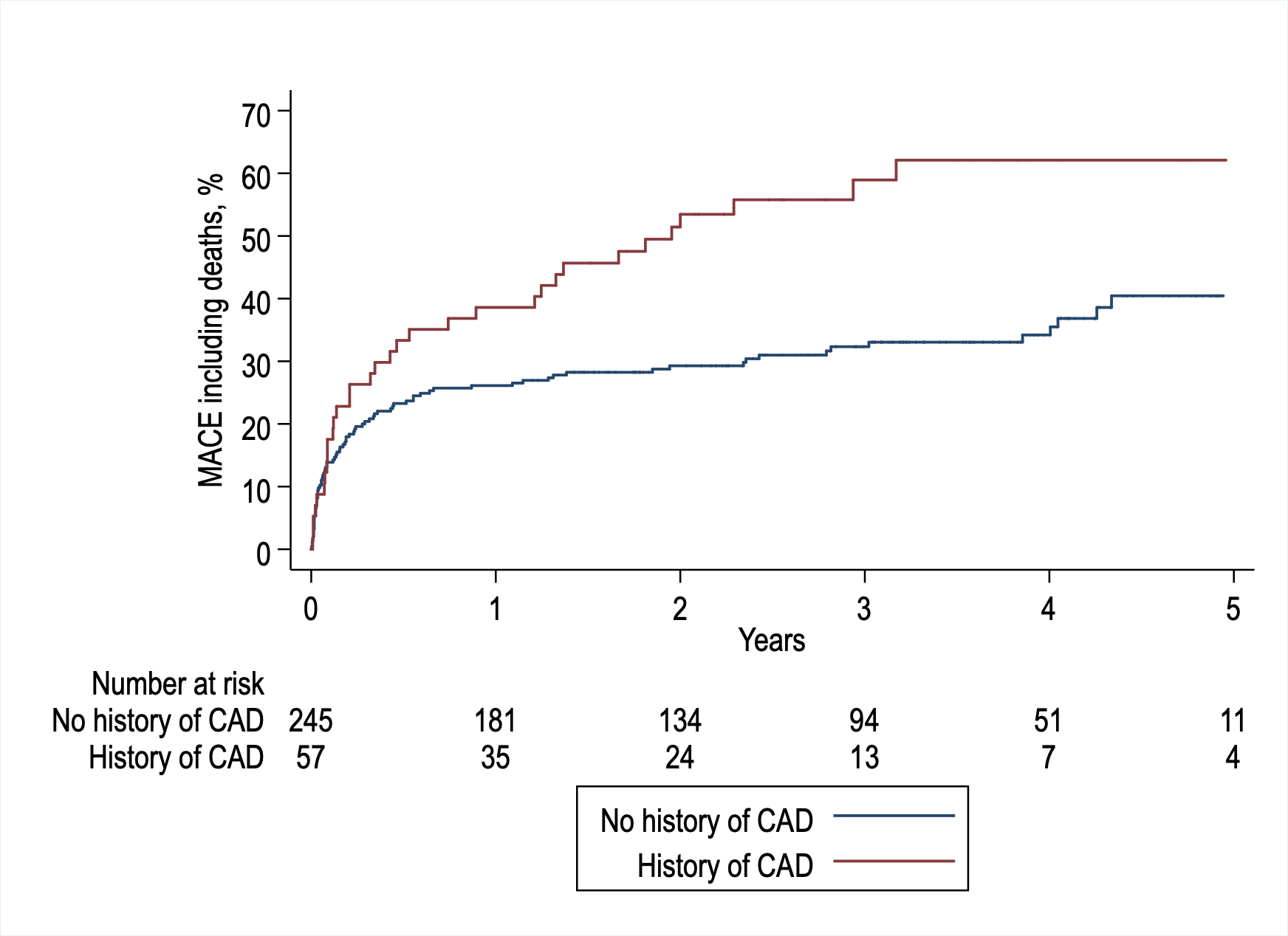
**

*B. Occurrence of major event according to patients with and without history of heart failure*

**
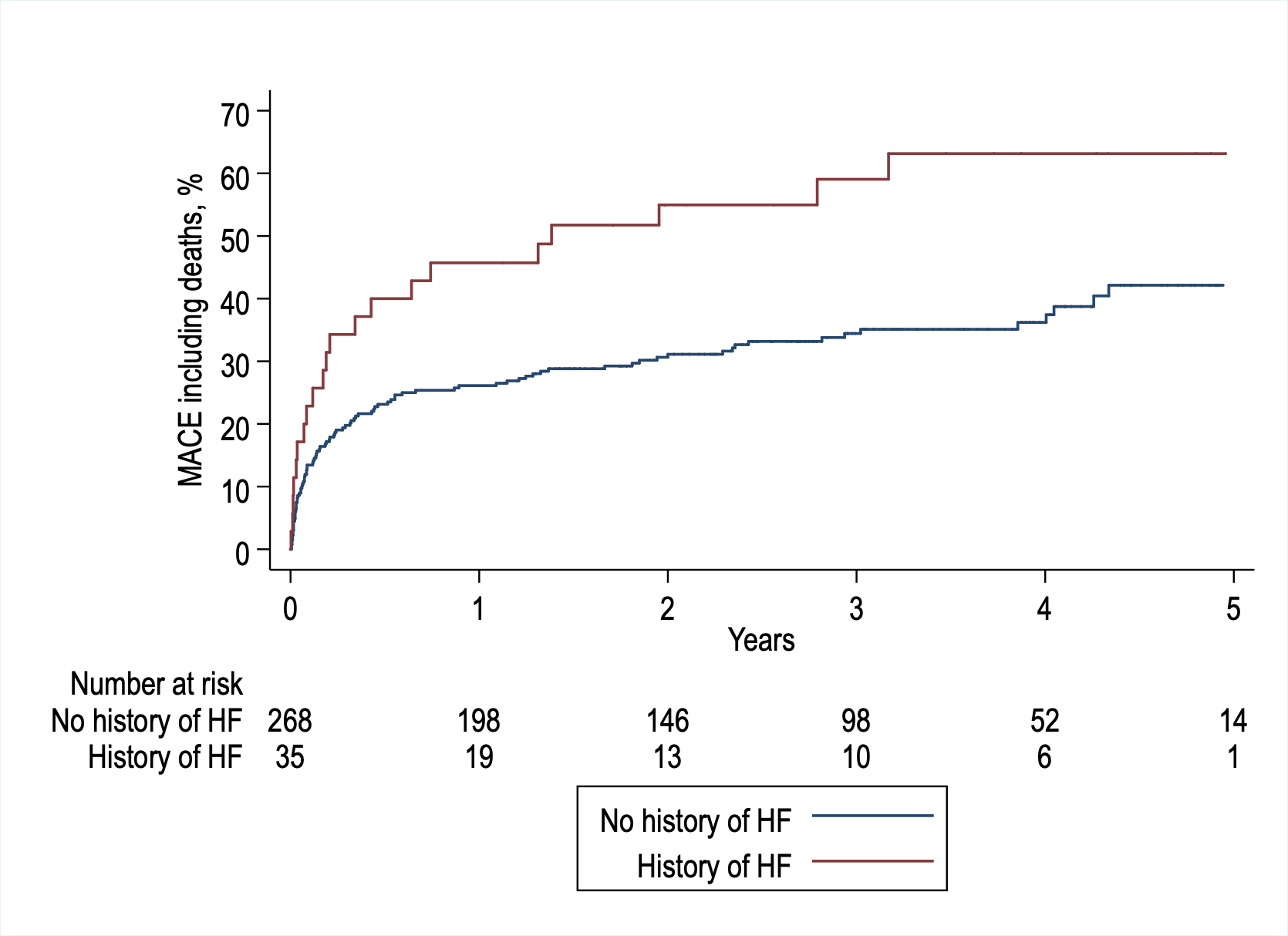
**

*C. Occurrence of major event according to patients with or without cardiac cause of OHCA*

*
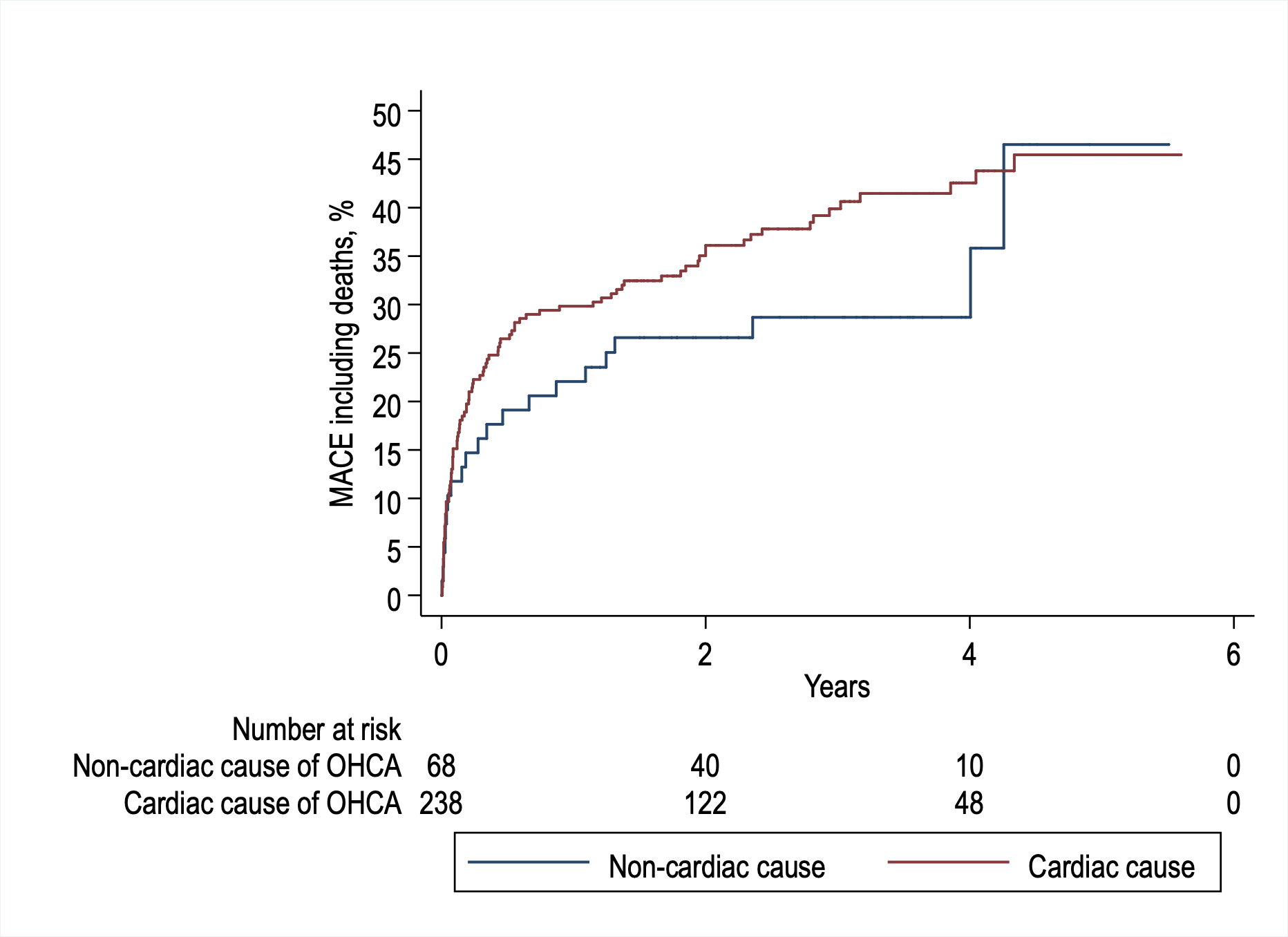
*

*OHCA out-hospital cardiac arrest*

*D. Occurrence of major event according to neurological status at hospital discharge (patients with CPC 1,2 or CPC 3,4)*


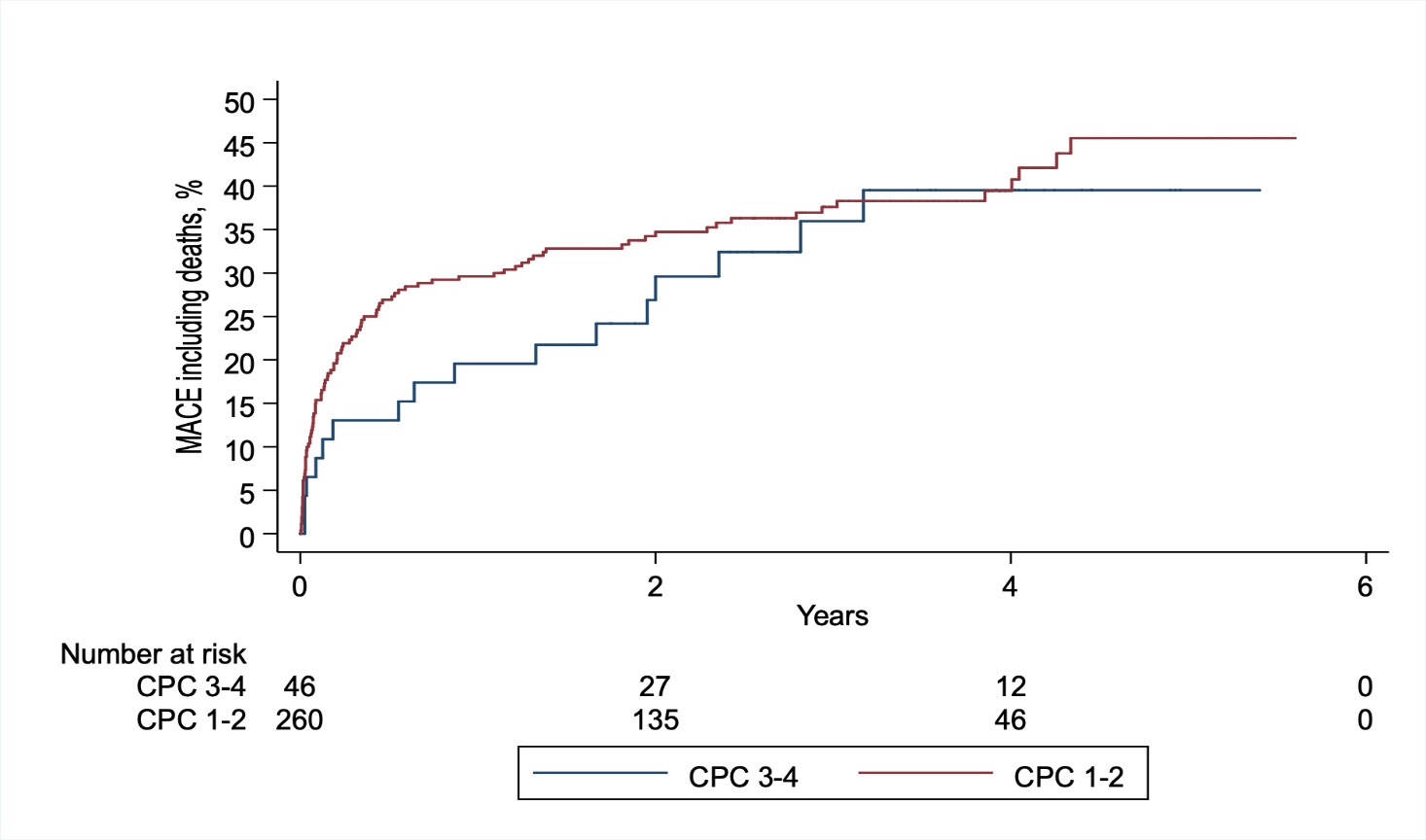

Supplement: Supplementary file 1 — Supplementary Material 1 [file 13613_2024_1371_MOESM1_ESM.docx]
